# Unilateral Condylar Hyperplasia With Active Bony Overgrowth: A Case Report

**DOI:** 10.7759/cureus.19059

**Published:** 2021-10-26

**Authors:** Hassan A Alsayegh, Zahraa A Alsubaie, Abdullah R Alwayil, Mukhtar A Alqadhi, Ali M Alawadh

**Affiliations:** 1 Department of Radiology, King Fahad Hospital, Hofuf, SAU; 2 Department of Oral and Maxillofacial Surgery, King Fahad Hospital, Hofuf, SAU; 3 Department of Nuclear Medicine, King Fahad Hospital, Hofuf, SAU

**Keywords:** bone scintigraphy, dental malocclusion, facial asymmetry, temporomandibular joint, condylar hyperplasia

## Abstract

Condylar hyperplasia (CH) is an overgrowth disorder of the temporomandibular joint bones caused by growth center overactivity of the mandibular condyle. Although the disorder is mostly idiopathic, several etiologies have been proposed. CH presents as progressive facial asymmetry with functional abnormalities. A combination of clinical, histopathological, and radiological findings is crucial to determine the diagnosis. Several diagnostic algorithms have been described in the literature. Management of CH is variable and depends on the growth of the bone. Here, we present the case of a 36-year-old female who presented with progressive facial asymmetry which was diagnosed as unilateral CH with active bony overgrowth.

## Introduction

Condylar hyperplasia (CH) is an overgrowth disorder of the temporomandibular joint (TMJ) bones that is caused by growth center overactivity of the mandibular condyle [1]. Growth overactivity of the mandibular condyle, which includes the condylar neck, head, and lower arch corpus, results in functional and cosmetic abnormalities such as the development of dental compensation, occlusal variability, and facial asymmetry because of the progressive overgrowth of the bone [2]. Clinically, CH presents as a progressive facial asymmetry and regularly occurs during the active growth phase, especially during adolescence [3,4]. The facial asymmetry caused by CH has been shown to have a diverse presentation [5].

Epidemiological data reviews have shown female predominance revealing a possible role of estrogen [3,5]. However, some studies have shown a similar incidence in males and females, as well as unilateral and bilateral mandibular CH [6,7]. Several theories have been proposed regarding the etiology of CH, including metabolic hyperactivity, endocrine alterations, genetic factors, trauma, joint infection, hypervascularization of the mandibular condyle, and intrauterine changes. However, the exact etiology remains idiopathic [8-10]. To distinguish CH from other facial abnormalities, proper clinical evaluation, histological findings, and imaging findings are crucial. Clinical assessment can be confirmed by nuclear medicine studies, particularly bone scintigraphy, to show the sites of abnormally increased uptake in the affected bone that demonstrates osteoblastic activity. Nuclear studies can be performed using bone radiopharmaceutical agents such as technetium 99m methyl diphosphonate (Tc-99m MDP) [10,11].

The facial asymmetry that occurs due to CH has distinct management. The activity of the condylar growth determines the choice of surgical intervention. If the condyles show active growth, the management becomes even more varied. For the active growth form of CH, condylotomy is an optimal surgical intervention [12-14]. If the imaging of CH shows an inactive growth form, the remnant facial asymmetry can be corrected via osteotomy of the mandibular joint. These surgical corrections restore good adjustment between both sides of the face, resulting in better function of the TMJ [15]. CH can influence the quality of life of patients as it affects swallowing, chewing, and phonetic abilities due to occlusal deformities. In addition, CH may cause nasal obstruction due to turbinate hypertrophy and deflection of the nasal septum [14,16].

Overall, CH is a self-limiting disorder. However, if the disease remains active, the discrepancy between both sides of the face progresses with the associated occlusal deformity [17]. Here, we present a case of unilateral CH in a 36-year-old female patient with active bony overgrowth of the mandible.

## Case presentation

A 36-year-old female presented to the maxillofacial surgery clinic complaining of gradually developing asymmetry of the right side of the face for the past 15 years. The progressive asymmetry of the entire right side of the face was noticed by her family members. Mandibular deflection toward the healthy side and overgrowth were noticed 15 years before, progressing gradually until they reached the present proportion. Furthermore, there was progressive development of pain in the TMJ region while opening the mouth. There was no history of trauma, vascular collagen diseases, infections, or surgery on the face or jaws. Her medical history and family history were noncontributory.

The extraoral examination confirmed facial asymmetry due to the downward displacement of the entire right mandible and an increase in the vertical height of the middle and lower facial thirds on the right side. The chin had deviated to the right side, and the lip line had slightly shifted downward, tilting toward the right side. Furthermore, mild tenderness was noted in her TMJs bilaterally, a clicking sound was heard during movement of the left TMJ, and tenderness was noted in the right side of the neck. The intraoral examination revealed a slight shift of the mandibular midline to the right side along with a mandibular cant. In addition, she had a posterior open bite, that is, posterior teeth of both the jaws were slightly tilted lingually to maintain the occlusion.

**Figure 1:**
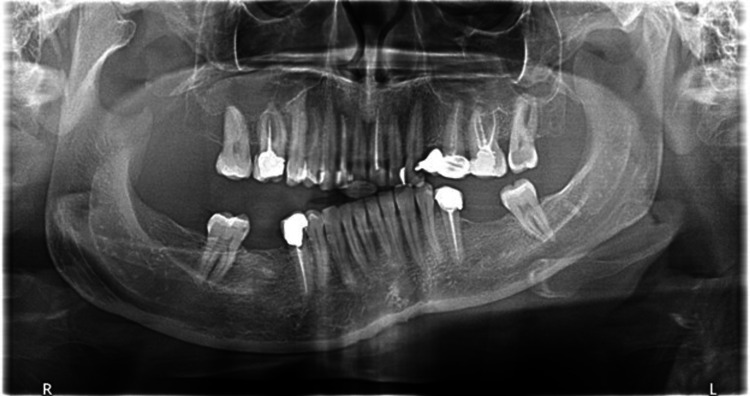
Panoramic radiograph of the mandible demonstrating an asymmetric enlargement of the right mandibular condyle and elongation of the right mandibular neck. Dental malocclusion on the right side can be seen.

**Figure 2:**
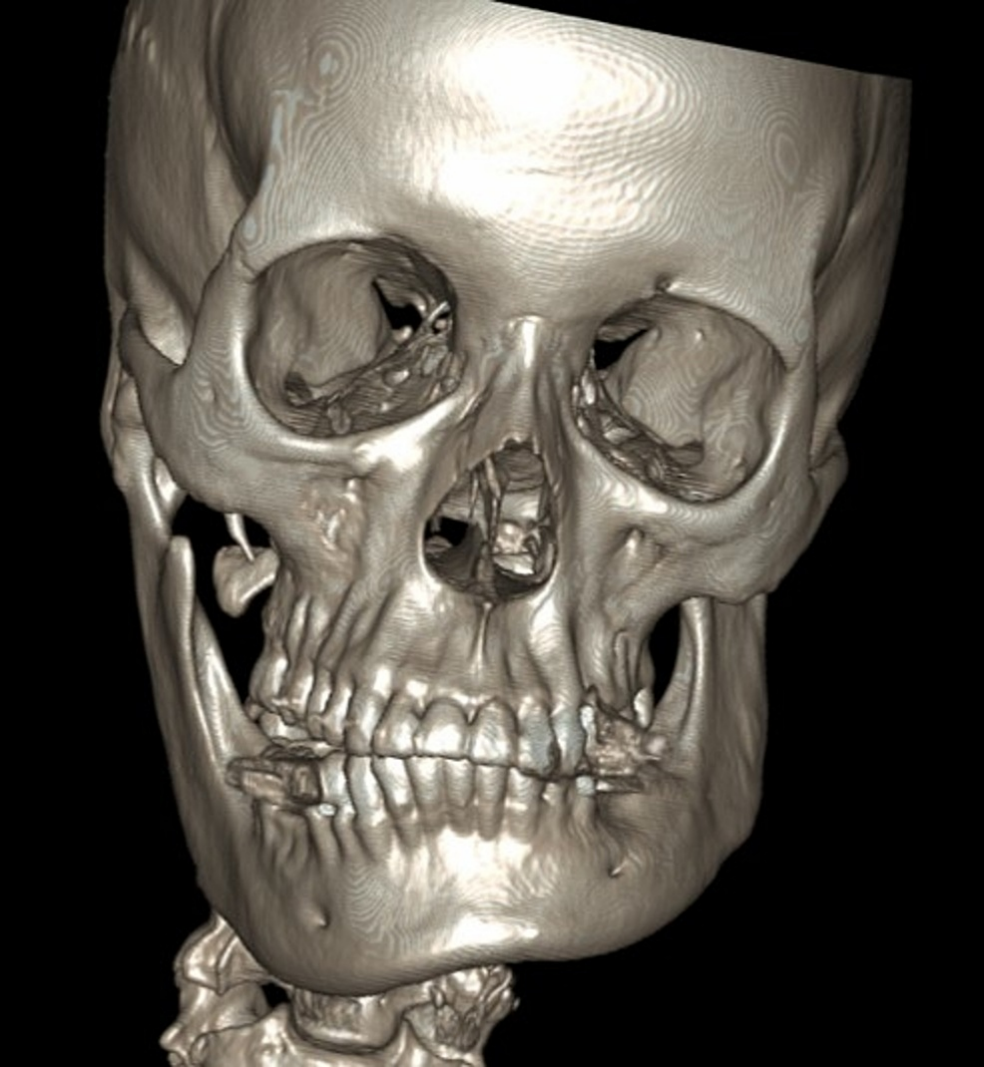
3D volume-rendering CT of the face showing asymmetry of the mandible. 3D: three dimensional; CT: computed tomography

**Figure 3:**
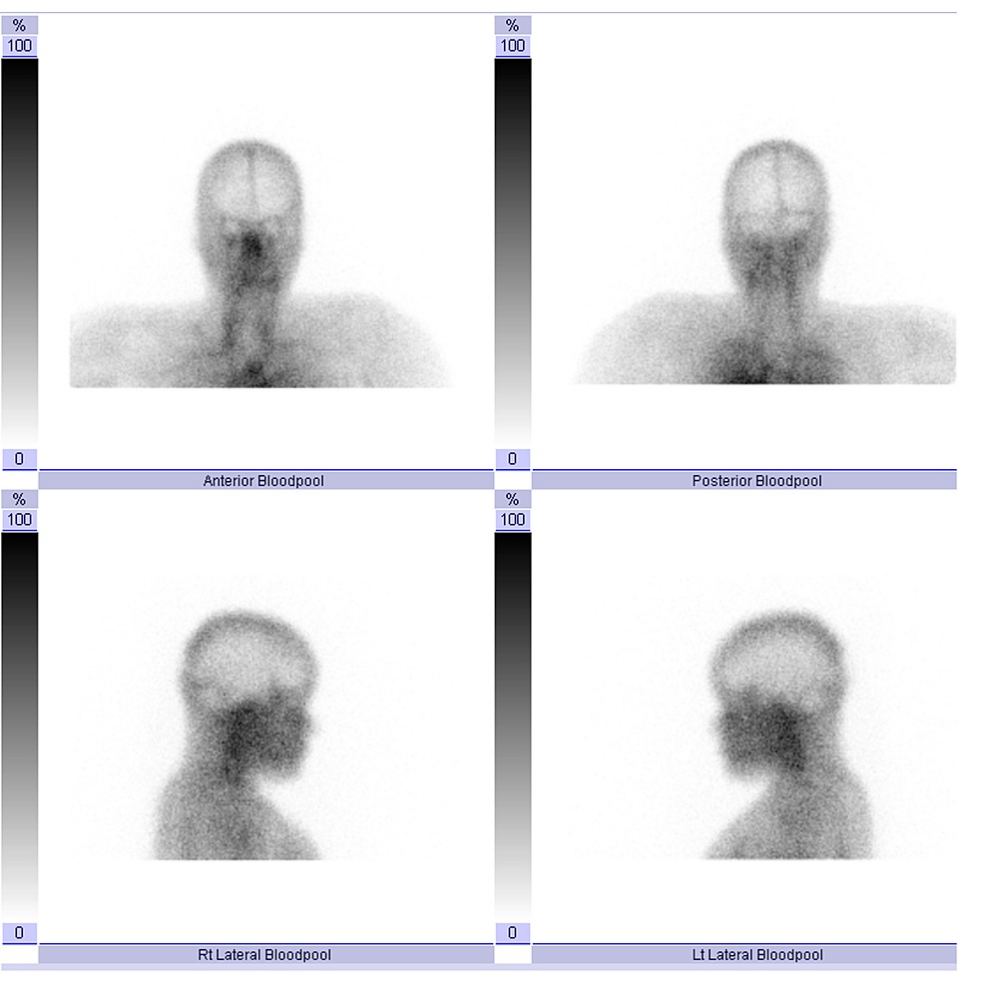
Three-phase bone scan. Blood pool images over the head are unremarkable.

**Figure 4:**
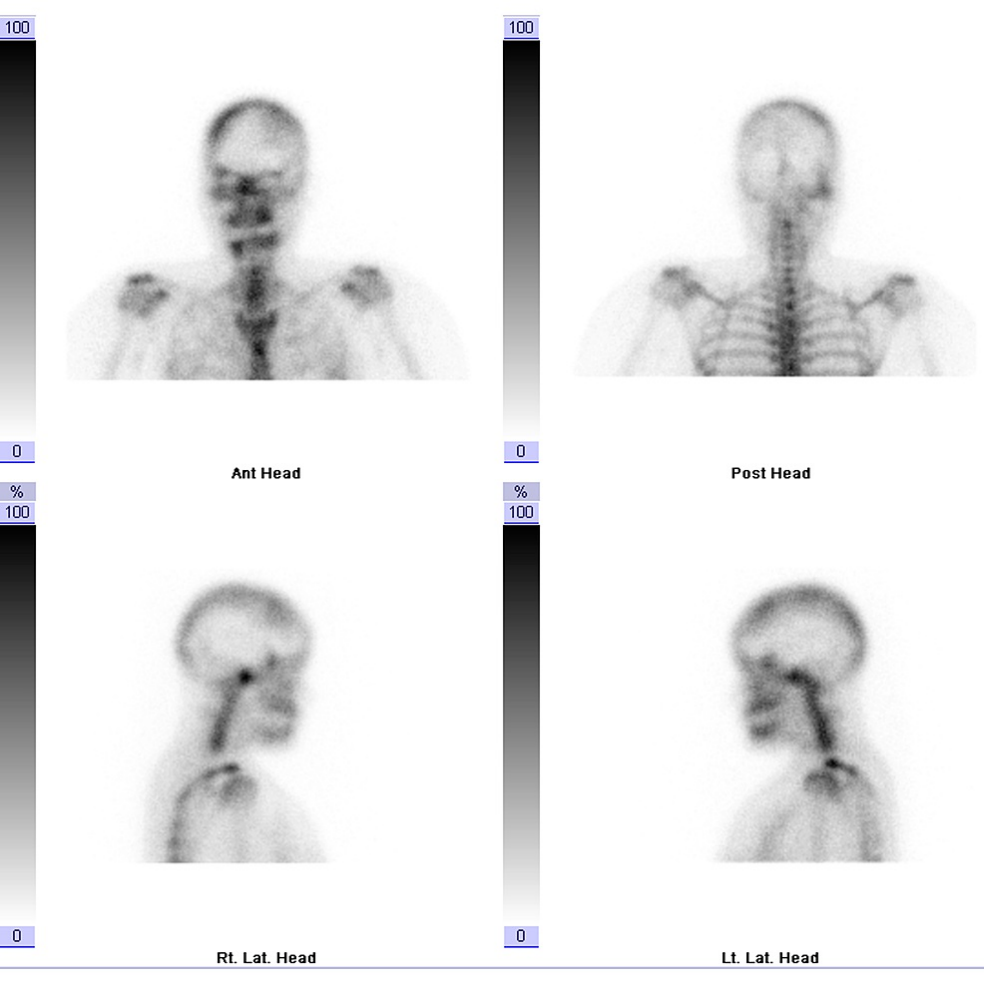
Three-phase bone scan. Three-hour delayed planar images demonstrating markedly increased focal tracer uptake at the right temporomandibular region. Bilaterally asymmetrical increased focal tracer uptake localized to the maxilla and mandible can be seen, related to dental pathological process, as confirmed on SPECT. SPECT: single-photon emission computed tomography

The panoramic radiographic image revealed significant uniform asymmetrical enlargement of the right mandibular condyle and elongation of the neck of the right mandibular bone, along with improper dental occlusion of the right side. The right gonial angle was characteristically rounded off and the mandibular canal was displaced to the lower border of the right mandible (Figure 1). Computed tomography (CT) scan was performed to further characterize the abnormality. Three-dimensional (3D) volume-rendering CT showed asymmetry and differences in the size of both condylar heads as well as elongation of the neck of the mandibular condyle on the right side (Figure 2). Accordingly, a three-phase bone scan along with single-photon emission computed tomography (SPECT) was performed to determine the activity of the condylar heads. The three-phase bone scan was unremarkable in blood flow (not shown) and blood pool (Figure 3) images over the head region. However, three-hour delayed planar images demonstrated markedly increased focal tracer uptake at the right temporomandibular region (Figure 4). Furthermore, there was bilaterally asymmetrical increased focal tracer uptake localized to the maxilla and mandible, which was related to the dental pathological process.

**Figure 5:**
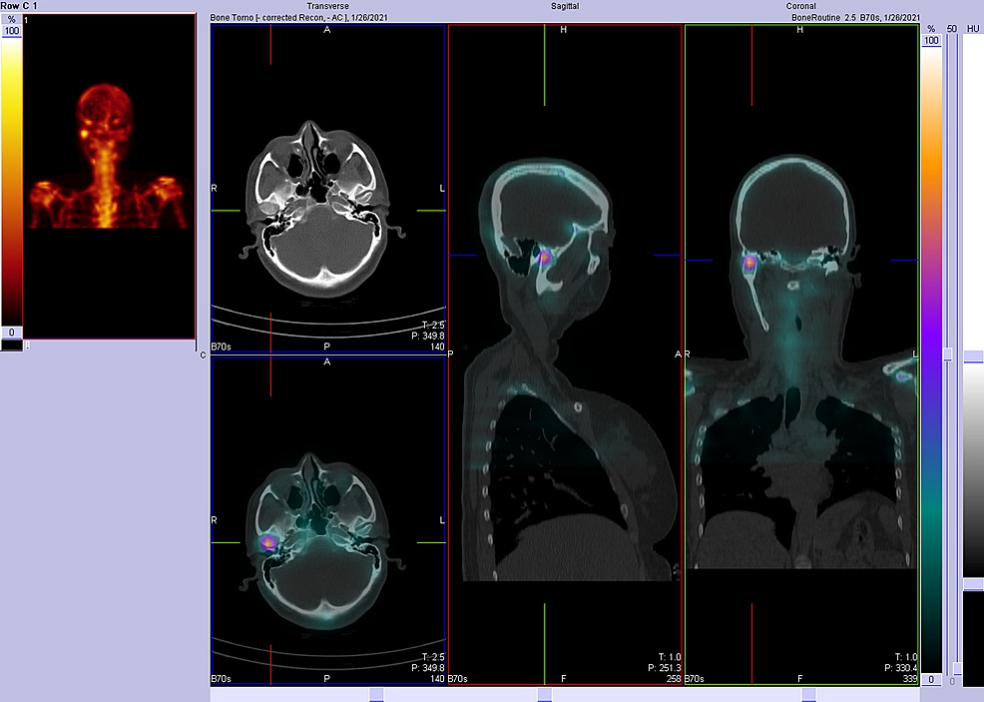
SPECT image demonstrating intense focal activity that corresponds to the right mandibular condyle which appears enlarged. It is associated with elongation of the right mandibular neck and tilt of the mandible to the left side. SPECT: single-photon emission computed tomography

**Figure 6:**
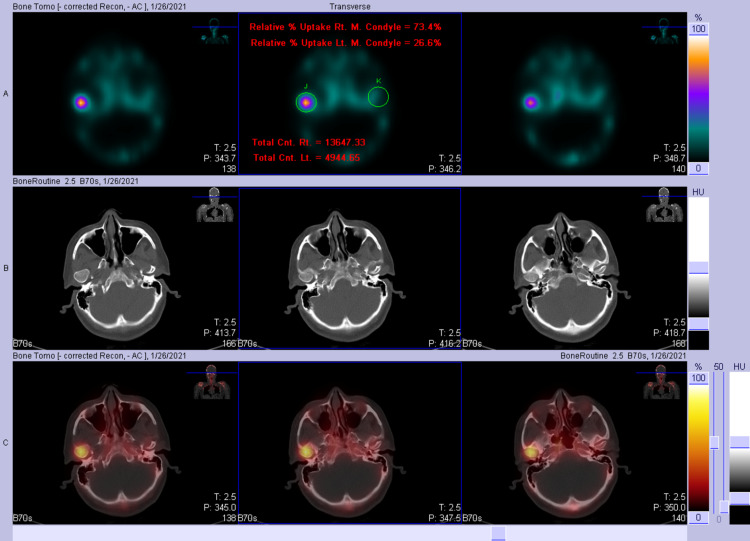
SPECT image demonstrating intense focal activity at the right mandibular condyle and normal physiological tracer uptake at the left mandibular condyle. SPECT: single-photon emission computed tomography

Thereafter, SPECT demonstrated intense focal activity corresponding to the right mandibular condyle, which appeared enlarged and measured 1.9 × 1.5 cm compared to the left mandibular condyle which measured 1.6 × 0.6 cm in transverse and anteroposterior dimensions. It was associated with elongation of the right mandibular neck and tilt of the mandible to the left side (Figure 5). The left mandibular condyle demonstrated normal physiological tracer uptake. The relative uptake of the right mandibular condyle was markedly increased at 73.4%, while the relative uptake of the left mandibular condyle was only 26.6%. There was no evidence of erosive or sclerotic changes at the TMJ bilaterally (Figure 6). Moreover, there were no erosive or sclerotic changes at the TMJs bilaterally.

The clinical and radiologic findings were consistent with a diagnosis of unilateral CH of the right side of the face. The patient was referred to a tertiary center to be managed accordingly.

## Discussion

In 1986, Obewegeser and Makek categorized the different imaging types of CH under two main categories: hemimandibular elongation and hemimandibular hyperplasia. In hemimandibular elongation, facial asymmetry is always found in the horizontal plane, whereas hemimandibular hyperplasia causes asymmetry in the vertical plane. In addition, the third type of CH is a combination of both classifications [17,18]. In hemimandibular elongation, the condyle remains intact, but the neck is overwhelmingly slender and distorted where the ramus is elongated. Hence, the first type of CH is termed hemimandibular elongation [19]. On the other hand, hemimandibular hyperplasia is linked with the hyperactivity of the prechondroblastic cells of the mandibular condylar joint. Hence, in the literature, cases of laterognathia are incorrectly included with horizontal condylar hyperplasia, which may explain the superiority of such cases being reported in the literature [20]. In the combination-type CH, there is an increase in the length of the lower part of the face on the affected side.

CH is related to the entire three-dimensional hemimandibular enlargement, ranging from ipsilateral mandibular condylar joint to the mandibular symphysis [5,21], with a sloping rima oris and minimum deflection of the chin to the other side of the face. Moreover, there is a deflection of the midpoint of the lower jaw toward the healthy side of the face intraorally, whereas the mandibular molars on the contralateral side demonstrate displacement lingually to remain in a suitable occlusion with the maxillary molar teeth. If the molars on the healthy side are unable to adapt to this unusual growth, it can result in a crossbite because of paradoxical growth between the lower and upper jaws [18,22]. Malocclusion that results from this bony overgrowth may be an important feature as occlusal crossbite abnormality impacts the condylar joint. Overall, due to the exaggerated downward abnormal growth of the mandible, the maxillary molars on the impacted side usually compensate by following the abnormal downward growth of the mandible [18]. Furthermore, maxillary alveolar bone on the affected side grows to preserve the occlusion of the teeth. This leads to a downward sloping on the occlusal plane of the affected side of the face [23]. If the maxillary molars cannot follow this extremely abnormal downward growth, the affected side will result in an open bite [18].

In the case of combined hemimandibular elongation and hemimandibular hyperplasia, the occlusal plane becomes oblique, resulting in an open bite if the maxilla is unable to follow this rapid abnormal growth activity. The chin becomes extremely deviated and the midpoint of the mandible moves toward the unaffected side, resulting in a crossbite on the healthy side [18].

In terms of the etiology of CH, the precise cause remains idiopathic. According to the literature, there is a contradiction among studies regarding gender predominance, as some authors reported that females and males are equally affected, while others emphasized a female predominance [22,24]. For instance, a retrospective study of 61 patients showed that females are more generally affected than males with a ratio of 3:1 [21], in contrast to other studies that did not find a female predominance [17].

Identification of the mandibular CH is based on a clinical and radiological assessment to estimate the growth activity of the joint and the dysmorphic features of the joint [17].

The radiologic classification system of facial asymmetry in CH was suggested by Obewegeser and Makek in 1986. CH is evaluated on CT scans with 3D reconstruction [17]. CT images with 3D reconstructions play a role in the evaluation of mandibular CH and to determine the exact radiological type [25]. However, according to one study, it varies and fails to follow the classification system of this growth abnormality [21]. Moreover, a limitation has been reported in terms of the utilization of SPECT, in which it cannot differentiate between CH and other conditions such as neoplastic, infective, inflammatory, or ongoing healing processes. This emphasizes the need to recognize the disorder with careful clinical assessment and dedicated anatomical and functional imaging [26]. Additionally, in patients with a history of rheumatoid arthritis or systemic lupus arthritis, there is a reduction in the condylar volume which may affect the diagnostic predicament [20]. In contrast, SPECT can provide 100% sensitivity and specificity for identifying abnormal growth hyperactivity in the condylar joint through an analysis of proportional percentage uptake activity of Tc-99m MDP [23]. Clinical evidence has been established that a difference of 10% or more between both condylar joint uptake is diagnostic for abnormal active growth of the disease [11,27]. Evaluating condylar hyperactivity is a crucial factor in determining the success of treatment [6]. Identification of active CH should not be limited to clinical workup and imaging studies. Alternatively, a combination of clinical follow-up, histopathological analysis, and bone scintigraphy should be used. Periodic follow-up in a 6-12-month interval has been suggested [25].

Regarding CH management, when the condyles show active growth, the management becomes more diverse. For active growth of CH, condylotomy is an adequate surgical intervention. Low condylotomy is used for removing the tumors of the TMJ [12-14]. Once the imaging of CH shows inactive growth, the residual asymmetry can be corrected by mandibular osteotomy. Ultimately, these surgical corrections were proposed to restore good adjustment between both sides of the face with improved function of the TMJ. Mandibular osteotomy can result in a good outcome where there is minimal compensatory growth of the maxillary dentoalveolar complex has been reported [15].

According to our experience in this case, the clinical and radiological data were sufficient to diagnose CH. The management was not included due to the referral of the patient to a tertiary center.

## Conclusions

CH is characterized by facial asymmetry in clinical settings. In the literature, controversy exists regarding diagnostic algorithms. However, a combination of detailed clinical and dedicated radiological diagnosis is crucial to diagnose CH in addition to periodic follow-up. Clinicians should consider CH in case of functional or cosmetic facial abnormalities. Furthermore, imaging findings play a vital role in diagnosing CH. Scintigraphy images are an assessment tool rather than a diagnostic tool to avoid an incorrect diagnosis. Other local or systemic conditions that may affect the diagnosis of CH during management should also be considered. Clinical and imaging follow-up is suggested in case of uncertain diagnosis. The surgical options to treat CH are variable and depend on the mandibular growth activity.
